# Copper/Epoxy Joints in Printed Circuit Boards: Manufacturing and Interfacial Failure Mechanisms

**DOI:** 10.3390/ma12030550

**Published:** 2019-02-12

**Authors:** Philipp Nothdurft, Gisbert Riess, Wolfgang Kern

**Affiliations:** 1 Chair of Chemistry of Polymeric Materials, Montanuniversität Leoben, A-8700 Leoben, Austria; gisbert.riess@unileoben.ac.at (G.R.); wolfgang.kern@unileoben.ac.at (W.K.); 2 Polymer Competence Center Leoben GmbH, Roseggerstraße 12, A-8700 Leoben, Austria

**Keywords:** copper/epoxy joints, state of the art, printed, circuit boards, failure analysis, electronic industry

## Abstract

Printed circuit boards (PCBs) have a wide range of applications in electronics where they are used for electric signal transfer. For a multilayer build-up, thin copper foils are alternated with epoxy-based prepregs and laminated to each other. Adhesion between copper and epoxy composites is achieved by technologies based on mechanical interlocking or chemical bonding, however for future development, the understanding of failure mechanisms between these materials is of high importance. In literature, various interfacial failures are reported which lead to adhesion loss between copper and epoxy resins. This review aims to give an overview on common coupling technologies and possible failure mechanisms. The information reviewed can in turn lead to the development of new strategies, enhancing the adhesion strength of copper/epoxy joints and, therefore, establishing a basis for future PCB manufacturing.

## 1. Introduction

**Figure 1:**
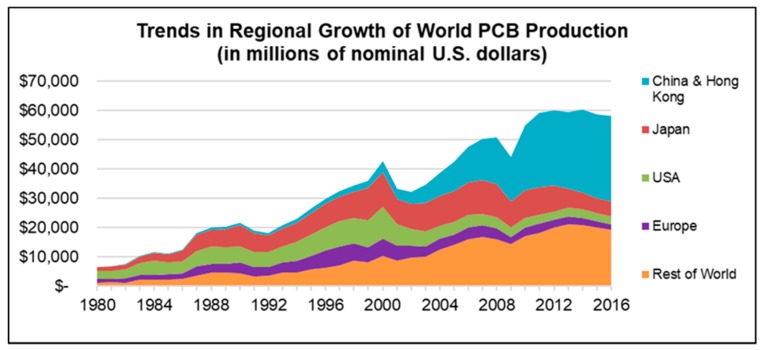
Trends in regional growth of world PCB (printed circuit boards) production (1980–2016). Reprinted with permission from [1]; Copyright 2016 IPC.

Nowadays, printed circuit boards (PCBs) are used in most electronics, ranging from cell phones, consumer electronics, automotive, etc. to high-end products such as sophisticated computer systems. The demand for PCBs has increased constantly, leading to an entrenchment of PCBs from the 1970s in nearly all branches of electronics. Since then, the electronic industry has experienced rapid development. In 2014, the worldwide PCB production value was estimated at 60.2 billion US dollars (Figure 1) and is still growing [1].

The invention of multi-layer boards triggered the miniaturization of electronic products and continued to drive PCB manufacturing technology towards smaller and more densely packed boards with increased electronic capabilities. Thereby, manufacturing is dependent on the adhesion between copper and epoxy composites. Due to increasing component density in PCBs and decreasing line width of copper wires and interconnects, the temperature within an electronic device can reach up to 200 °C during operation [2]. Weak copper/epoxy joints cause failures during the application of multi-layer boards. Crack growth at the interface of the copper/epoxy joint and subsequent delamination are the consequences. In addition, when advancing to thinner copper foils, finer copper patterns or application in the high frequency sector, the type of bonding between copper and epoxy resin is of high importance. Improving the adhesion between the copper and the polymeric backing is crucial in providing better performance, resistance to cracking and delamination and, therefore, higher reliability.

## 2. Manufacturing of Copper/Epoxy Joints

The state-of-the-art processes for surface pretreatment of copper in PCB manufacturing are divided into etching and chemical bonding technologies and are discussed in the following chapters. The copper/epoxy joints are formed by laminating a pretreated copper with epoxy resin composites, and these are the major insulating materials that are used in PCB manufacturing under heat and pressure.

### 2.1. Etching Technologies

The need for processes to promote adhesion between individual circuit board layers arose as multi-layer boards became a high volume product in the 1970s and the complexity of circuit board design increased. Different technical paths were explored during that time, all attempting to solve this problem. Initially, these pre-treatments were based on roughening the copper surface in a chemical etching process. The surface topography and area of the metal significantly influences the adhesion strength and, as a consequence, the mechanical bond [3,4,5]. The processes, based on etching, create a textured copper oxide surface with or without an additional organic coating which allows the formation of mechanical interlocks during the pressing/lamination step to epoxy resins. More than 95% of the adhesion strength arises from the increased surface area and roughness that is generated during etching [6]. The resulting mechanical bonds are extremely strong and have a high resistance to hydrolytic and thermal degradation [7].

#### 2.1.1. Black Oxide

Since the early days of the PCB industry, the adhesion improvement between organic prepreg layers and copper foils was performed using a process known as “black oxide treatment”. This technique has long been the market leader for inner-layer bonding of advanced materials and is still used in appreciable volumes today [8]. Advantages of the black oxide process are (i) the increased available surface area for bonding through the rough, porous copper oxide crystals, resulting in high peel strength and, most importantly, (ii) thermal reliability due to superior mechanical bonding and passivation of the copper to prevent corrosion during the manufacturing processes at elevated temperatures [9].

The black oxide process consists of several chemical steps, which at the beginning remove native oxides and organic contaminants from the copper surface, followed by the formation of carefully grown nano-scale oxide features [10,11,12,13,14]. The characteristic black color of the copper foil after treatment gives the process its name. The artificially created copper oxide layer is formed during immersion of copper substrates in a bath containing the following components [12,14,15,16]:

- oxidizing agent (e.g., sodium chlorite, potassium persulfate),
- sodium hydroxide,
- electrolyte (e.g., trisodium phosphate or sodium sulfite).

**Figure 2:**
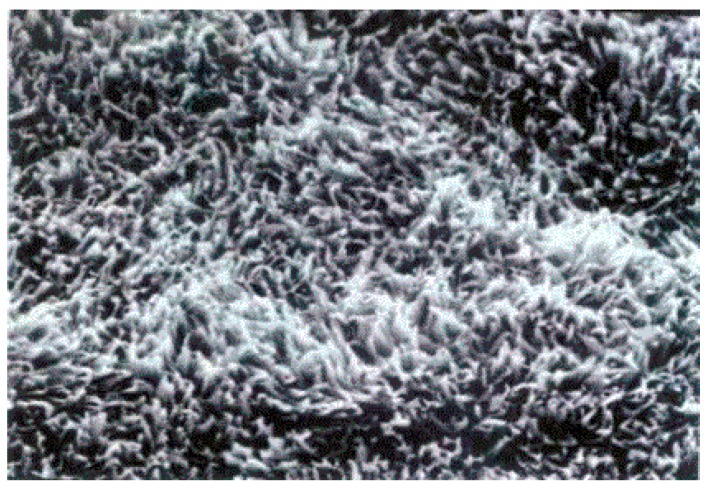
Scanning electron microscopy (SEM) image of a 10k× fold magnification black oxide treated surface. Reprinted with permission from [17]; Copyright 2002 Atotech GmbH.

The bath is adjusted to temperatures between 65 and 95 °C. This type of chemical oxidation forms a surface topography as shown in Figure 2 and is responsible for the mechanical interlocking at nano scale with the thermosetting resin. 

Within the first minute, smooth pebble like precipitates of cuprous oxide (Cu_2_O) are formed on the surface and coarsened to the average size of 0.2 µm. In that state, the surface roughness is not altered. The formation of a Cu_2_O layer becomes inactive when fibrillar CuO starts to nucleate after 60 seconds. On the top of the pebble-like facets, acicular precipitates of cupric oxide (CuO) of 0.5 to 1.0 µm in length start to nucleate as the metallic copper surface gets depleted and grows more or less constantly over the whole surface. The growth of acicular cupric oxide dramatically increases roughness before saturation [9]. The surface morphology remains unchanged with further oxidation, while the cupric layer increases in thickness [9,14,18].

The binding strength between the epoxy resin and the black oxide coating correlates with the precipitation of CuO nucleated on the smooth Cu_2_O layer [19]. The Cu_2_O layer has no significant effect on adhesion, while the acicular CuO crystals increase the toughness proportionally with thickening of the CuO layer up from 0.1 to 0.3 µm. Once a continuous layer of CuO crystals is formed, the adhesion cannot be further strengthened. Maximum bond strengths can be achieved after treatment between 3 and 5 min [9]. Yun et al. also stated that the black oxide process has a minimum thickness of coverage without defects and the probability of failure within the oxide layer increases with exceeding a specific minimum copper oxide thickness (100 µm) [12]. 

Exposing black oxide/epoxy resin specimens to oxidizing solutions (e.g. KMnO_4_ solution in the desmear process for drill hole cleaning) results in the removal of epoxy material next to the interface. Therefore, the black oxide treated surface is much more prone to attack by subsequent acidic solutions. Thus, desmear processes in combination with follow-up acidic treatments are detrimental to the bond durability of black oxide/epoxy joints [12,15,20]. The consequence is partial delamination, mostly occurring around drilled through holes of the PCB board. This phenomenon is called “pink ring” because of the removal of the black oxide surface and the exposing of the bare copper surface which appears pink under the microscope. The occurrence of pink rings is an indication of poor quality control of the manufacturing process by the PCB manufacturer. The columnar fibrillar cupric oxide crystals are also very fragile and easily fractured during multiple lamination cycles or when not handled carefully [17]. The “bleeding” influences the downstream manufacturing processes as well as the conductive integrity of the board. Improvements were made to avoid the unwanted removal of loosely bound oxide crystals and to enhance the acidic resistance of the black oxide coating. The “converted oxide” or “reduced oxide” process was evolved. Thereby, an additional step of the process is introduced which reduces the surface cupric oxides of the fibrillar structure back to Cu^0^, enhancing the toughness and acid resistance of the crystals. Nevertheless, the black oxide treatment is quite time consuming, uses aggressive agents, such as sodium hydroxide or sodium chlorite at temperatures near boiling point of the solution and demands high energy consumption [14]. The limitation of the black oxide technology became apparent and, as a consequence, was superseded by the commonly known “oxide replacement technologies”.

#### 2.1.2. Oxide Replacement Technologies

**Figure 3:**
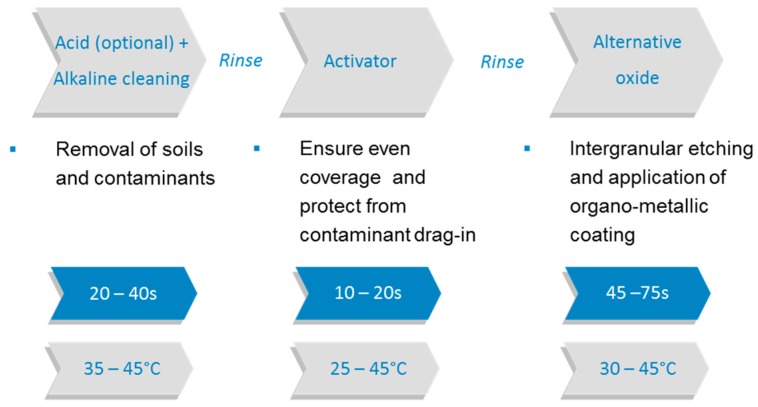
Schematic flow chart of the alternative oxide process.

The oxide replacement technologies were developed in the mid to late 1990s due to high energy consumption, weak reliability in acid environment and the vertical processing conditions of the black oxide process [15]. Nowadays, they are widely seen as the “standard” adhesion promoting processes and are marketed under different trade names (Top bond promoter 7878-JKEM, Oxidestar–Wisecompany, CZ-2030–Uvemura, Bondfilm–Atotech, etc.). Although it is not possible to achieve peel strengths equal to the black oxide process, the overall advantages, such as thermal reliability, excellent protection against pink ring and wedge voids, stable surface profile, optimized chemical resistance, sufficient inner layer adhesion, high capacity horizontal processing, short treatment time, simple chemical control, etc., ensured the process changeover to alternative oxide processes in multilayer board production [8,17]. Unlike their predecessor, the surface roughness is not achieved by growing copper crystals on the surface of the circuit, however by etching down into the copper. Thus, a very dense microstructure is provided by a modified sulfuric/peroxide etchant (intergranular etch) to ensure mechanical bonding between the copper and the polymeric material. Mechanical adhesion is still predominant. In addition, an organo-metallic coating is also formed by reaction with cyclic amine compounds (e.g., benzotriazole derivatives) which gives some level of chemical adhesion between the treated copper and the applied dielectric material (e.g., epoxy resin) [21,22]. Figure 3 shows a general overview of the entire oxide replacement process.

**Figure 4:**
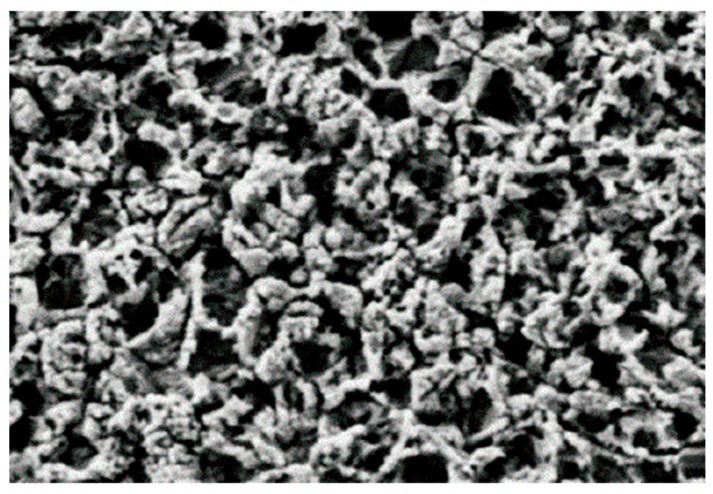
5000× fold magnification of an alternative oxide treated surface by SEM measurement. Reprinted with permission from [17]; Copyright 2002 Atotech GmbH.

The characteristics of the process are the microetching of copper to a depth of 1.2 to 1.5 µm, while at the same time converting the copper at the surface (200–300 Å) to the organo-metallic structure. During the rinsing and drying steps, the copper surface is modified within approximately three minutes in the conveyorized (horizontal) mode and leads to a structured copper surface (Figure 4).

In the following, a brief overview of the chemical reaction mechanism and characteristics of the alternative oxide treatment is given. The general reaction for the etching is considered to be:

(1) $${\mathrm{Cu}+H}_{2}{\mathrm{SO}}_{4}{+H}_{2}O_{2}\to {\mathrm{CuSO}}_{4}{+2H}_{2}O$$

**Figure 5:**
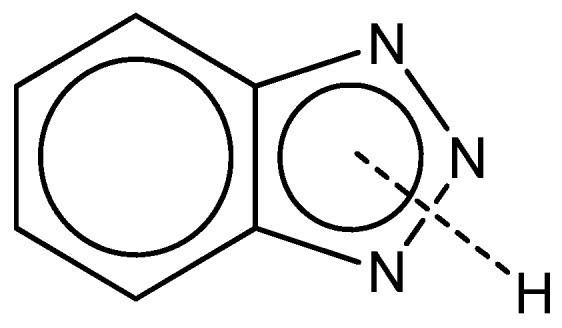
Resonating form of BTAH (benzotriazole).

This reaction is performed nonspecifically throughout the surface, therefore no structured surface is obtained. The addition of benzotriazole (BTAH) or a derivative of BTAH to the treatment bath is responsible for the texturing of the surface and the formation of an organo-metallic coating. BTAH is an organic molecule consisting of a benzene and a triazole ring (Figure 5).

The chemical structure of BTAH reveals the ability to act as a weak acid by releasing a proton or as a base by accepting a proton to one of the nitrogen atoms. Thus, it can exist in three forms (BTAH^+^, BTAH and BTA^−^) depending on the pH value of the solution [23]. The lone pair of nitrogen atoms and the aromatic ring structure allows the formation of a coordinative bond to the copper surface. In 1963, Dugdale and Cotton observed that the inhibition efficiency of BTAH is due to true chemical bonding to metallic copper or its metal oxides [24]. Thereby, the orientation of the molecule is reported as vertical or tilted [25,26] or almost parallel to the surface [27,28]. Nageh et al. argues that BTAH molecules arranged vertically or tilted can exercise lateral interactions to each other, giving a higher degree of surface coverage [29]. In contrast, Antonijevic and Petrovic favor the parallel orientation because of the formation of stronger bonds due to the interaction of π-electrons of the ring with the d-orbitals of copper [30].

The general chemistry of BTAH adsorption on copper is described in the following: 

In the first step, BTAH is adsorbed on the copper surface

(2) $${\left[\mathrm{BTAH}\right]}_{\mathrm{aq}}↔{\left[\mathrm{BTAH}\right]}_{\mathrm{ads}}$$

where [BTAH]_aq_ refers to BTAH in the aqueous phase and [BTAH]_ads_ to that of adsorbed species. The equilibrium of (2) is shifted toward the right by increases of the BTAH concentration in the solution. As a result, a higher degree of surface coverage is obtained. A further reaction results in the formation of a protective film of Cu(I)BTA complex (where BTA is the anionic form of BTAH):

(3) $$\mathrm{Cu}+{\left[\mathrm{BTAH}\right]}_{\mathrm{ads}}\to \left[\mathrm{Cu}(I)\mathrm{BTA}\right]{+H}^{+}{+e}^{-}$$

Here, the copper oxidation is mediated by H_2_O_2_ as the oxidizing agent (see Equation (1)). Many researchers have given ample evidence that the Cu(I)BTA complex exists in a polymeric form [27,31,32],

(4) $$n\mathrm{Cu}(I)\mathrm{BTA}\to {\left[\mathrm{Cu}(I)\mathrm{BTA}\right]}_{n}$$

while Youda et al. proposed that adsorption and complex formation are in equilibrium [32]

(5) $$n{\mathrm{BTAH}}_{\mathrm{ads}}+n\mathrm{Cu}\to {\left[\mathrm{Cu}(I)\mathrm{BTA}\right]}_{n}+nH^{+}+ne^{-}$$

The reaction mechanisms reveal that both increasing the pH value and the concentration of BTAH in solution favor the formation of a [Cu(I)BTA]*_n_* protective polymer complex. At lower inhibitor concentration and in acidic media, adsorption becomes favorable. BTAH also reacts with CuO surfaces, however the rate of adsorption onto a Cu_2_O surface is more rapid [33]. The etching and, as a consequence, the release of Cu^2+^ ions in a solution results in [Cu(II)-azole]_2_ complexes. These complexes are only partially soluble in the system and, therefore, develop precipitates. This may lead to bath maintenance problems and the requiring of filtration. Nowadays treatment baths have a copper holding capacity of at least 28–45 g/L [34]. Typically, adsorption of BTAH is an exothermic process. Thus, performing the process at elevated temperatures would have an adverse effect on the inhibition efficiency and film formation [35]. What is most problematic for treatment baths containing BTAH and its derivatives is the presence of chloride ions. Investigations by Modestov et al. described the influence of chloride ions on the Cu(I)BTA complex formation [31]. At high anodic potential, corresponding to oxidation of the copper surface, a thin CuCl film is formed between Cu_2_O and the Cu-BTA complex. Due to mechanical tension related to the appearance of a CuCl interlayer, local ruptures of the Cu-BTA film occurs. It is also possible that the reason for the removal of the Cu-BTA film is a poor adsorption between Cu-BTA and CuCl layers. This behavior causes severe problems for the alternative oxide process, reducing the stability and reliability of PCBs. Thus, the avoidance or the addition of an additive to withstand a certain amount of chloride ions is mandatory.

**Figure 6:**
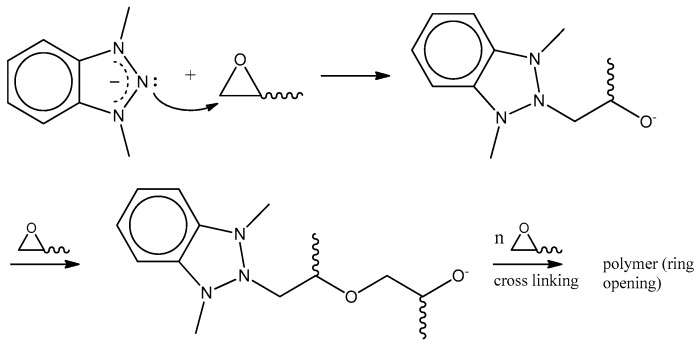
Curing of epoxy resins with BTAH.

Pure BTAH is known as a useful cross-linking agent for epoxy resins [13,21] and the reaction mechanism proposed by Xue et al. is shown in Figure 6 [21].

The benzotriazol-copper complex initiates a ring opening of the epoxy functionality. The formed adduct can subsequently react with another epoxy group and initiate anionic polymerization. As already stated, the use of BTAH derivatives is also quite common. It was reported that derivatization has no effect on the inhibition mechanism while it influences the inhibition efficiency [36,37]. Moreover, the bonding to epoxy resins significantly depends on the introduced organic residue [12,38,39]. Beside enhancing the performance of the treatment bath by modification of the inhibitor molecule, synergistic effects on the inhibition performance are reported for BTAH and thiourea, sodium dodecyl sulfate or other components [28,40].

Summing this up, the alternative oxide process baths are solutions of BTAH or its derivatives, peroxides, sulfuric acid and different additives. This results in the characteristic organo-metallic surface with a defined structure and the economical sustainability of the process bath. Nevertheless, the principle is simple and consists of texturing the surface with simultaneous adsorption of a robust passivation layer. However, the roughening nature of the process will eventually be rendered obsolete in the production of future PCBs [41].

### 2.2. Chemical Bonding Technologies

Up until now, technologies to attach copper foils to the epoxy resin have mainly been based on a chemical roughening of the metal surface. During chemical roughening, the copper foil is etched in the micrometer range which essentially constitutes the limitation of this process.

A copper etching of approximately 1.0 µm is necessary to attain good adhesion with the prepreg materials. Proceedings towards thinner copper foils or patterns finer than 10 µm lines and 10 µm spaces were limited by the roughness obtained in the etching step. In addition, a rough surface is disadvantageous in the high-frequency sector. At operating frequencies approaching the GHz range, the electrical current density is largest near the surface and decreases with greater depths in the conductor. This phenomenon is known as the “skin effect”. Any small changes in surface area or roughness result in a significant impact on the impedance and, consequently, in signal loss [42,43]. Thus, it is important to achieve surfaces as flat as possible with only minor copper etching.

#### 2.2.1. Non-Etching Adhesion Promoters

**Figure 7:**
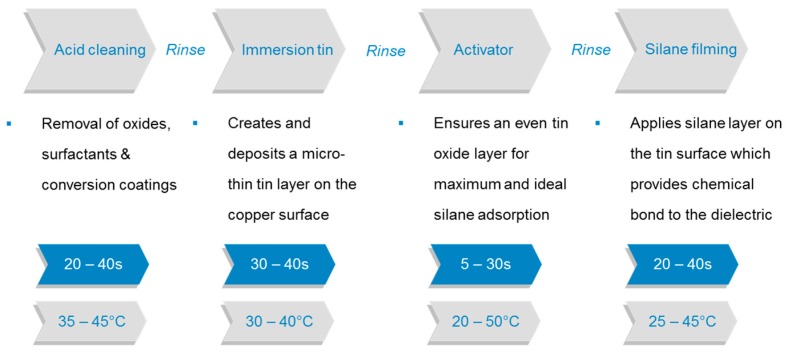
Schematic representation of the white oxide process.

Although the oxide replacement technologies are seen as the standard processes for PCB manufacturing, the earliest alternative oxide techniques were based on non-etching adhesion promoters and are commonly known as “white oxide”. The name is derived from the use of metals being deposited on the copper surfaces in order to facilitate adhesion to organic molecules by decreasing the isoelectric point (IEP) of the surface [8,44]. Typically, these metal conversion layers consist of a thin tin layer (<0.1 µm) and the subsequent coating of an organosilane which further reacts with the prepreg systems. The process steps of the white oxide process are shown in Figure 7.

Since tin is less noble than copper, there is no replacement reaction between Cu^0^ and Sn^2+^ occurring because the electrode potential of copper is more positive than for tin (electrode potential −0.14 V for Sn^2+^ ↔ Sn^0^ and +0.52 V for Cu^+^ ↔ Cu^0^). Typically, there are two ways of electroless Sn deposition technologies on copper. In an autocatalytic procedure, a tin layer is deposited as a result of a disproportionation reaction in an alkaline solution.

(6) $$2{\mathrm{Sn}}^{2+}\to {\mathrm{Sn}}^{4+}+\mathrm{Sn}$$

A uniform tin layer can only be achieved when it is deposited on an already existing tin surface on copper. Thus, a two-stage procedure is necessary. In the first step, a thin tin film is precipitated on copper followed by the autocatalytic plating step to deposit a smooth and uniform tin layer [45,46]. Due to the high hydrogen overpotential of tin, the applicability of this process is limited. Hence, hydrogen-containing reducing agents (e.g., sodium hypophosphite) cannot be used for electroless tin deposition.

Thus, the attention was directed to the utilization of the tin immersion procedure. It is characterized by a displacement reaction of copper by tin.

(7) $$\mathrm{Cu}+{\mathrm{Sn}}^{2+}\to {\mathrm{Cu}}^{2+}+\mathrm{Sn}$$

This reaction does not occur spontaneously. The potential for both reactions changes when a very strong copper complexing agent (thiourea) is added. The Cu^+^/Cu potential becomes lower than that of Sn^2+^/Sn, resulting in electroless tin deposition on copper. Once a thin film of tin is formed, the replacement reaction begins to limit itself until a certain film thickness is reached, however it may continue in the presence of reducing agents [47,48,49,50].

A typical tin plating bath requires:

- Sn^2+^ source (e.g., SnCl_2_, SnSO_4_),
- Complexing agent (thiourea),
- Acid (e.g., H_2_SO_4_, HCl, methanesulphonate, buffer solution),
- Temperature (30–60 °C),
- Optional: additives (e.g., reduction agent, bath stabilizers).

After deposition of a thin film, the activator step is introduced to fully convert all available tin surfaces into oxides and/or hydroxides. A tin oxide/hydroxide surface serves as a convenient anchor for further chemical modifications (e.g., (3-glycidyloxypropyl)trimethoxysilane) forming stronger bonds between the copper surface and the epoxy resin systems.

The white oxide processes were the first commercially successful, large-scale, conveyorised oxide alternatives for multilayer bonding and are still listed in the product portfolio of well-known companies (Secure HFz–Atotech; Flat Bond–MEC). It provides superior chemical bonding to most resins without roughening the copper surface, however it lacks in mechanical bonding which limits its use when resin systems do not form chemical bonds and rely more on mechanical adhesion. In addition, concerns were raised because of the presence of tin. Pure tin and tin alloys (e.g., Cu_6_Sn_5_) have the potential of forming whiskers and dendrites under specific conditions [51,52] and can cause short circuits and arcing. Thus, the deposition of a thin tin layer is not appreciated and cannot fully replace the present state of the art black oxide process. Despite that, the idea behind the white oxide processes has become the basis of the next generation of adhesion promoters.

#### 2.2.2. Azole Derivatives

**Figure 8:**
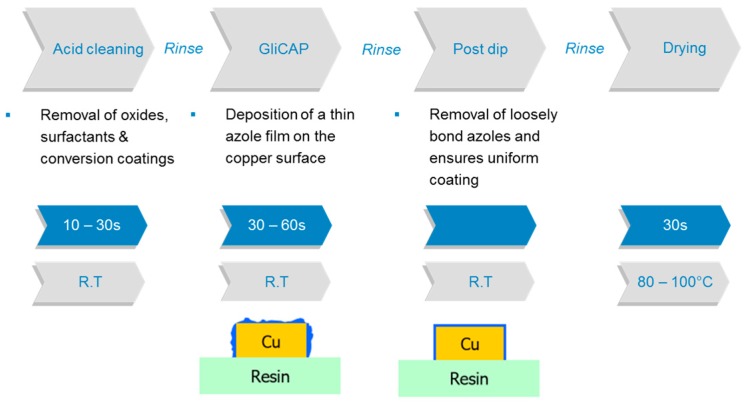
Process steps for the deposition of a thin azole silane film on copper.

A new route to follow is based on a particular azole derivative acting as an interfacial adhesion promoter. The attachment to the copper surface is possible because of the azole group, whereas a functional group, specifically silanol groups, interact with the epoxy resin (“Glicap technology” by Shikoku company) [53]. The idea behind this technology is not new. Many researchers focused on copper modification with silanes [54,55], phosphonates [56,57], thiols [58,59], imidazoles, azoles [60,61], azole derivatives [62,63], etc., however none of the bonds were sufficiently thermally stable (e.g., Cu-S, Cu-Si, Cu-P) nor was an up-scaling of the treatment possible. Here, the reinstatement value of the process is attributed to the synthesis of specific azole silane derivatives at a larger scale and its economic feasibility. A uniform thin organic film (approximately 10 nm) without pre-treatment of the copper surface (e.g. with tin) is deposited, resulting in excellent adhesion to the insulating materials for innerlayers without roughening the copper surface and the compatibility with various prepreg materials, and is also non-epoxy based with high glass transition temperature *T*_g_. The coating procedure is shown in Figure 8:

This technology also has the advantage that the copper/epoxy laminate can be CO_2_-laser drilled and the resin with the azol-coating can be removed during the soldermask developing solution [64]. The adhesion improvement was similar to the etching type technologies. The organic layer does not influence the electrical reliability (surface insulation resistance or electro migration) or the plating (via filling) or finishing processes (nickel over gold). However, the disadvantages are almost the same as for the white oxide process. The silanol functional group forms bonds with many different prepreg systems, with some exceptions, thus limiting the applicability of this process to specific prepreg types. In addition, the azole modification agent has to be synthesized and is not commercially available from sources other than the providing company which affects the price of the Glicap process.

## 3. Investigations on Interfacial Adhesion Failure

**Figure 9:**
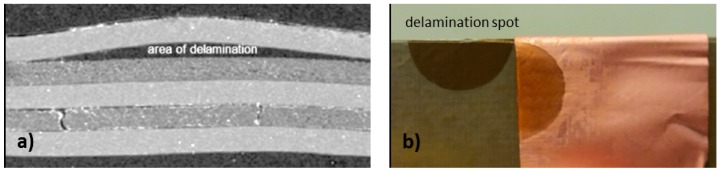
Cu/epoxy delamination failures after reliability testing. An SEM image of the cross section (**a**) and a photographic image after peeling (**b**) are shown.

The term “interphase” was coined by Sharpe and describes the transition zone when dissimilar materials are brought into contact [65]. Many researchers have investigated the interfacial regions between metal substrates and various polymers. Because of the requirements for strong adhesion in PCBs and other electronic applications (e.g., integrated circuits substrates), investigations on adhesion and failure modes of copper/polymer interfaces are mandatory. The strength of the bond after lamination usually predicts the performance in reliability tests and the long-term stability. An initially weak interface leads to delamination failures (Figure 9).

Different surface modification techniques have been evaluated over the years to find alternative processes to common binding techniques. Besides, many researchers have put effort into analyzing the Cu/polymer interface and to understand possible causes of delamination failure. In the following paragraphs, these theories are discussed.

### 3.1. Oxidative Degradation of Polymers

The oxidative degradation of polymers such as polyolefins [66], polyimide [67,68], polyesterimide enamel coatings [69], poly(ether ether) ketons [70], polyethylene [71], or epoxide resins [72] attached to copper substrates has been widely investigated. The formation of copper carboxylates and the diffusion of these salts [71,73] or other copper species [66,69] into the polymer matrix appears to have adverse effects on the stability of metal-organic systems. The catalytic role of copper metal in the sulfide formation at the copper/(poly)phenylene sulfide interface is responsible for almost the complete loss of peel strength [16]. McElhaney et al. described the oxidative degradation of poly(ether ether) ketons in high vacuum on cuprous and cupric oxide surfaces [70]. He proposed that the reduction of copper oxides is an integral part of the degradation process. Evans et al. stated that the good adhesion of copper to polyethylene is a consequence of the tendency of cupric oxide to react with metallic copper at 200 °C, forming a thermodynamically more stable cuprous oxide film in the absence of polymer oxidation [11]. In addition, it was reported that the cuprous/cupric ion pair is an effective oxidation/reduction couple that accelerated the decomposition of polymers [74,75].

### 3.2. Weak Boundary Layer

The effect of Cu_2_O and CuO ions on Cu/epoxy interfaces is not only affecting the oxidative degradation, however also the curing reactions. When an epoxy resin is mixed with an amine curing agent, competitive adsorption may take place when it is brought in contact with copper. The amine diffuses from the bulk of the resin to an immediate area near the surface [76]. High concentrations of amino groups near the copper surface deteriorate the adhesion because of the hydrophilic nature of the amino groups, stoichiometric imbalance or stiffness changes [77]. Van Ooij pointed out that the preferential adsorption depends on the metal surface and results in a thin polymer layer with reduced crosslinking density and low cohesive strength in close vicinity to the metal surface [78]. The change in curing mechanisms in the polymer boundary zone may result from these characteristics [79]. Miller et al. proposed preferential adsorption of dicyandiamide (DICY) to the copper oxide surface [80]. The depletion within the epoxy resin near the interface region leads to the decrease in polymer crosslinking density and overall adhesion. This is due to the competing reactions between DICY molecules and polymer hydroxyl groups (acid-base type bond formation to copper) for copper oxide atoms [81]. Even small amounts of cuprous and cupric oxides in the epoxy resin can increase the amount of unreacted DICY left in the cured resin, become spots for water absorption lowering the *T*_g_ and could act as stress concentration sites in the resin matrix [82,83]. Hong et al. demonstrated that primers coated onto a CuO surface prevent the adsorption of DICY and block the direct contact of CuO with epoxy resin [20]. A shift to higher activation energy is revealed by TGA measurements, indicating an increase in thermal stability and altered degradation mechanisms. Besides curing agents, low molecular weight fractions and additives are also concentrated at the interface, forming weak boundary layers [84].

### 3.3. Mismatches at the Cu/Cu_2_O/CuO Interfaces

Poor bond durability is also related to mismatches within the copper oxide layers. At ambient conditions, a thin film of cuprous oxide is formed on bare copper substrates since the Gibbs energy for the formation is a large negative number [85].

In general, the degree of oxidation will be a function of [86]:

- temperature,
- environmental conditions,
- heating duration,
- surface impurities or contaminants and the surface finish of the metal.

Further oxidation leads to the formation of cupric oxide on top [87]. Many researchers attributed the adhesion failure of Cu/polymer joints to the weakness of the CuO morphology [88,89] or instability within the oxidation layer [90,91].

**Figure 10:**
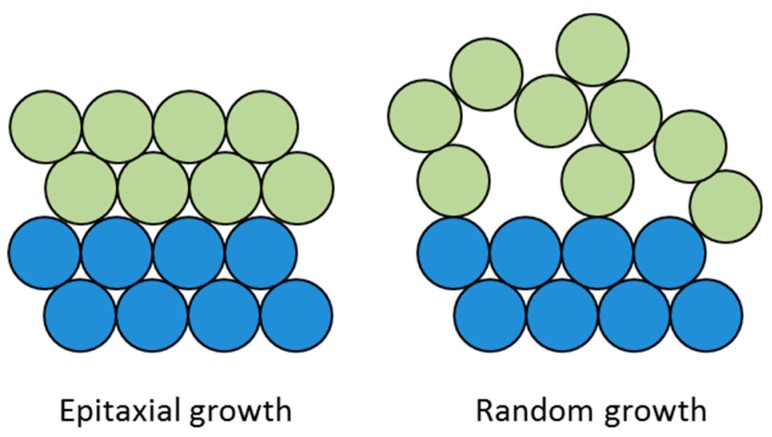
Schematic of crystal growth.

The growth of cuprous oxide is known by a specific epitaxial relationship at the beginning of oxidation, while random growth is favored for CuO with a highly asymmetric monoclinic crystal structure (Figure 10) [92]. The Cu_2_O/CuO interface with no epitaxial relationship generates internal stresses due to its different lattice structure and is much weaker than the Cu/Cu_2_O interface. The inherent brittleness and density difference between Cu_2_O and CuO is responsible for the weak interfacial adhesion with polymer resins [87]. At the initial stage of oxidation, the formation of an oxide layer increases the peel strength (<20 nm). Cho et al. reported that growth of oxide grains (originating either from Cu_2_O or CuO) changes the roughness of the surface so that epoxy resins undergo mechanical interlocking, which in return leads to higher peel values [91]. In accordance with thiss, an enhancement of button shear strength to epoxy was observed because of thin Cu_2_O/CuO formation during hygrothermal ageing on bare Cu [93,94]. The strongest interfacial adhesion with polymeric resins is achieved with oxide thickness between 20 and 30 nm, while further oxidation causes deleterious influence on the copper/polymer adhesion with significant reduction in peel strength [87,90]. The weak boundary of the Cu_2_O/CuO interface is responsible for adhesion failure when the oxide thickness exceeds 50 nm. The oxidation of copper metal at elevated temperatures is also occurring under the protection of coated films and decreases the adhesion strength [95].

### 3.4. Surface Tension, Wettability and Moisture Uptake

Moisture adsorption is one of the major reliability concerns in plastic packaging and PCBs because many failure mechanisms are believed to arise from the diffusion of water or water vapor during manufacturing, storage or operation [96,97,98]. The degrading effects of moisture lead to adhesion loss, hygroscopic swelling and vapor pressure formation at the interface when accelerated stress tests and solder reflow processes are performed. The presence of copper oxide species on the surface is accompanied by changes in surface tension and wettability of the substrate. The wettability is improved in the order Cu → Cu_2_O → CuO and the surface energy doubles from 30 mJ/m^2^ for bare copper to 70 mJ/m^2^ after Cu-oxide formation [9,91]. In the literature, a strong correlation between the difference in measured adhesion strength and surface energy (direct measurement of intermolecular forces) was found [9,93,99]. Kinloch stated that copper in its pure form exhibits poor adhesion characteristics for bonding to polymeric substrate because it is difficult to be wetted by adhesives [100]. The investigations by Lebbai et al. ascribe the main benefits of black oxide coating not only to enhanced mechanical interlocking, however also to passivation of the copper surface and improved wettability (copper oxide formation) [9]. Thus, the presence of a thin film of copper oxides is favored by enhancing the contact area between the substrate and various polymeric materials and, as a consequence, also enhancing the peel strength. 

The wettability of a surface also correlates with moisture uptake which in fact can have a huge impact on the adhesion strength. For plastic IC packages, a phenomenon known as “popcorn cracking” is based on adsorbed moisture within the packages’ various material interfaces. When exposed to normal ambient air (temperature, humidity), moisture diffuses into the molding compound and causes degradation of the adhesion strength. Water diffusion—in either liquid or vapor form—is due to polar groups that are present in the composite material [101,102]. Tencer proposed that vapor water molecules undergo phase transformation and condense to the liquid phase [103]. The condensed moisture was reported to be either in the form of discrete droplets on the surface or in the form of uniform layers. These water layers can act as weak boundary zones and reduce the adhesion between copper and epoxy resins. Micro cracks during reflow soldering allow moisture to enter the delamination gaps, resulting in a vapor pressure build-up. When the dooming exceeds the adhesion strength, package cracks occur [94,104]. A similar process is plausible within copper/polymer joints. While moisture is not able to diffuse through the copper substrate, the uptake of water is only possible through the bulk of the polymeric materials or via the substrate/adhesive contact area. The permeability of moisture through polymeric films and coatings depends on several factors such as crystallinity, hydrophilic/hydrophobic ratio, the polymer chain mobility, interaction between the film-forming polymer and the substrate, the presence of plasticizers, fillers or other additives, etc. In general, the hydrophobicity of bare copper substrates is advantageous with regard to lowering the affinity of moisture adsorption, the degree of oxidation under ambient environment and, subsequently, the susceptibility of the Cu/epoxy interface to debonding due to hydrolysis reactions [105]. On the other hand, a hydrophilic surface is required for adhesion promotion to prepreg systems.

If Fick’s law is applied, the permeability coefficient is described as the product of a thermodynamic parameter (sorption coefficient) and a kinetic parameter (diffusion coefficient) [106]. In general, the diffusion rate along the interface is faster than within the bulk adhesive which leads to a higher moisture concentration between the two materials [66,107]. For weak interfaces, crack growth was detected immediately as moisture appeared and slowed down the stronger the interface became [108]. Based on Shirangi and Michel, the moisture induced adhesion degradation is based on three mechanisms [107].

The first mechanism is related to an intrinsic aggregation effect of water molecules upon direct presence at the interface. The interfacial adhesion is degraded by bonding of water to the substrate or the polymer chains. For example, it was reported that moisture adsorption on a bare Cu surface is much faster than on a black oxide treated specimen, while the oxidation of bare copper at high temperatures and/or humid environments is directly related to the high retention of moisture and is mainly responsible for the degradation of adhesion [9,93]. It was also stated that thermal aging has an impact on the formation of additional polar functional groups into epoxy resins, which in turn increases the water adsorption. This, in turn, decreases the durability of the bonds [20].

The second mechanism correlates changes of the mechanical properties of polymeric materials to the adsorbed moisture [82,83]. The moisture uptake in the polymeric material causes a swelling which generates an additional mismatch in hygroscopic strain (volumetric expansion of metal and adhesives).

The third mechanism is due to volumetric mismatch between substrate and adhesive because of moisture induced swelling of the polymeric material.

### 3.5. Differences in Coefficient of Thermal Expansion

Other factors that weaken the adhesion strength of copper/polymer joints are the mismatch of the coefficient of thermal expansion (CTE) between the two materials and microvoid formation. Regarding the first point, the thermal stress, which is induced by the difference in thermal expansion coefficients between the different layers, induces high interfacial stresses and initiates cracks [109,110]. These cracks can subsequently propagate the delamination process. The incorporation of woven glass fibers in FR-4 materials significantly reduces the expansion in the in-plane direction (*x*,*y*), obtaining similar CTE values as for the copper substrates. The out-of-plane (*z*) expansion is not influenced by the reinforcement and causes stresses in plated through holes (barrel fracture, shoulder fracture and inner layer separation) [111].

### 3.6. Microvoid Formation

Moisture adsorption is one of the major reliability concerns in plastic packaging and PCBs because many failure mechanism are believed to arise from the diffusion of water or water vapor during manufacturing, storage or operation [96,97,98]. The degrading effects of moisture lead to adhesion loss, hygroscopic swelling and vapor pressure formation at the interface when accelerated stress tests and solder reflow processes are performed. In general, these complex failure modes arise from internal stresses and applied thermo-mechanical loads at the copper/epoxy boundary. The key factors which induce microvoid formation are not identified yet. Chong et al. observed internal void growth along the copper metal/oxide interface at 280 °C as the degree of oxidation is increased [112]. The weakness in adhesion of over-oxidized copper samples because of micro void formation was also proposed by Cho et al. [87]. Entrapment of air bubbles during the lamination process also results in the occurrence of voids. The adhesion performances of various resins are associated with the resin viscosity, gelation time and surface energy during curing [94,113]. While a low viscosity and a slow curing of epoxy resins results in good wetting of the copper surface, high viscosity polymers need a longer spreading time to achieve sufficient contact. The gelation time defines the available time frame. Fast curing of an epoxy resin with high viscosity leads to entrapment of air bubbles along the interface, giving rise to a weak interface.

## 4. Conclusions

The manufacturing of PCBs is a dynamic and constantly evolving industry, and the adhesion strength in copper/epoxy joints is of high importance for performance and reliability. Nowadays, binding technologies bear advantages and disadvantages for industrial and electrical demands, however they require development for future application. Thus, it is necessary to understand the phenomena which lead to delamination and crack propagation. The possible failure mechanisms indicate that delamination within copper/epoxy joints is a very complex phenomenon and depends on the characteristics of the prepreg systems (e.g., curing agent, viscosity, moisture absorbance, etc.), the copper surface (thickness of oxide layer, copper species) and interactions when both are brought into contact. Various theories have been developed to gain more information about interfacial failure mechanisms, however a predominant effect has not been found yet. Due to this review, a better understanding and the possibility for future development/technologies might be enabled.
